# Naringin Interferes Doxorubicin-Induced Myocardial Injury by Promoting the Expression of ECHS1

**DOI:** 10.3389/fphar.2022.859755

**Published:** 2022-04-12

**Authors:** Zirui Zhao, Shilei Yang, Yawen Deng, Liang Wang, Yifen Zhang, Zhenyu Feng, Han Li, Zhongchao Chi, Yunpeng Xie, Deshi Dong

**Affiliations:** ^1^ Department of Pharmacy, The First Affiliated Hospital of Dalian Medical University, Dalian, China; ^2^ College of Pharmacy, Dalian Medical University, Dalian, China; ^3^ Institute of Cardiovascular Diseases, The First Affiliated Hospital of Dalian Medical University, Dalian, China; ^4^ Department of Clinical Pharmacy, Huludao Center Hospital, Huludao, China; ^5^ Department of Critical Care Medicine, The First Affiliated Hospital of Dalian Medical University, Dalian, China

**Keywords:** doxorubicin, apoptosis, oxidative stress, inflammation, Naringin, ECHS1

## Abstract

Doxorubicin (DOX) leads to myocardial cell damage and irreversible heart failure, which limits the clinical application of DOX. Naringin has biological functions of inhibiting inflammation, oxidative stress and apoptosis. Our aim was to investigate whether Naringin could prevent DOX-related cardiotoxicity in mice. Naringin was administered by gavage and mice were intraperitoneally injected with doxorubicin (1 mg/kg/day) for 15 days. H&E, Masson, TUNEL and others experiments were examined. NRVMs and H9C2 cells were treated with Naringin and DOX *in vitro*. Using IF, ELISA and Western Blot to detect the effect of Naringin and ECHS1 on cells. The results showed that Naringin could prevent DOX related cardiac injury, inhibit cardiac oxidative stress, inflammation and apoptosis *in vivo* and *in vitro*. Inhibition of ECHS1 could interfere the effect of Naringin on DOX-induced myocardial injury. Naringin may provide a new cardiac protective tool for preventing the cardiotoxicity of anthracycline drugs.

## Introduction

In recent years, with the improvement of cancer diagnosis and treatment, cancer has gradually become a chronic disease, and the survival time of cancer patients has also been prolonged. But studies show that nearly half of cancer patients die from non-cancer events, with cardiovascular disease as the leading cause of death (Howlader et al., 2010; Jemal et al., 2010).

Currently, cancer treatment-related cardiotoxicity is mainly classified into two categories: type I and type II (Moudgil and Yeh, 2016). Type I chemotherapy-related cardiotoxicity refers to the presence of dose-dependent myocardial necrosis and large area of irreversible damage in cardiomyocytes after chemotherapy, which is mainly caused by anthracycline (Khouri et al., 2012). The mechanism is mainly positive correlation with cumulative dose, but the mechanism of myocardial injury has not been fully elucidated. At present, it is believed to be related to excessive production of reactive oxygen species (Antonucci et al., 2021), lipid peroxidation (Quagliariello et al., 2019), DNA damage (Qiao et al., 2020) and accumulation of tumor suppressor proteins (Li et al., 2019).

**FIGURE 1:**
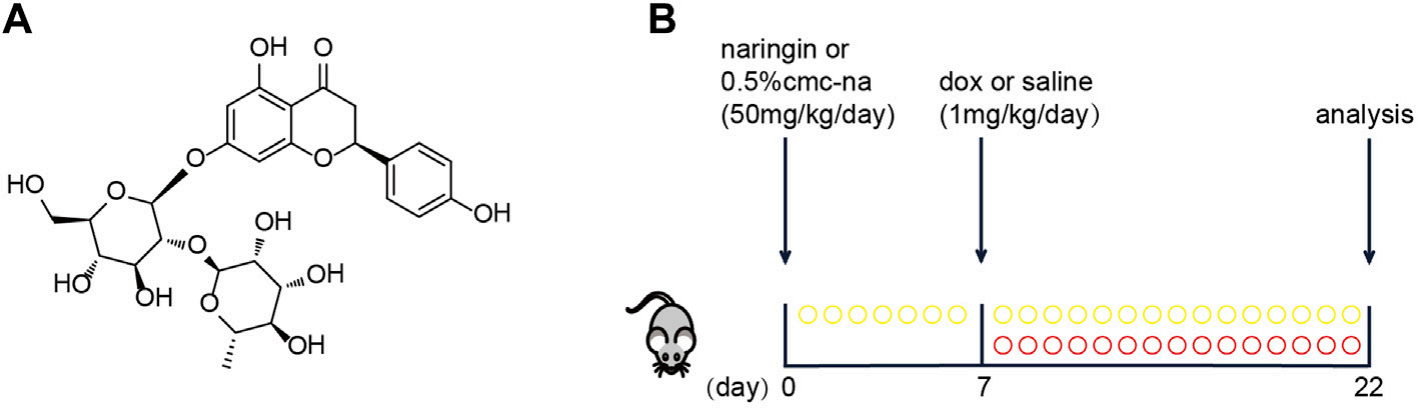
**(A)** Basic structure of Naringin (NR). **(B)** Schematic protocol for mice treatments showing models of exposure doxorubicin (DOX), which were performed in mice to test the efficacy of Naringin (NR).

Polyphenolic compounds (flavonoids, anthocyanins, and phenolic acids) are known for their antioxidant properties, free radical scavenging, and iron chelation properties (Balea et al., 2018). Naringin is a flavonoid that is a phenolic compound found in grapefruit and other citrus (Figure 1A). It has a variety of pharmacological and therapeutic properties, including anti-cancer, anti-inflammatory, and antioxidant effects (Raha et al., 2020). Naringin in myocardial infarction, Ischemia and reperfusion (Rajadurai and Prince, 2009), cardiac hypertrophy (Park et al., 2018) and other cardiovascular diseases play a role in cardiac protection. It has been reported that Naringin could protect doxorubicin-induced myocardial injury in rats (Jian et al., 2017), but it has not been involved in mice.

The mitochondrial short-chain enoyl-CoA hydratase encoded by the ECHS1 gene exists in the mitochondrial matrix as a 160 kU homohexamer. ECHS1 is involved in the second step of β-oxidation of short-chain fatty acids in mitochondria. Its role is to hydrate the trans-double bond between carbon 2 and carbon 3 in trans-2,3-enoyl CoA Forms L-3 hydroxyacyl CoA (Sharpe and Mckenzie, 2018).

In this study, the protective effect of Naringin and ECHS1 on doxorubicin-induced myocardial injury was found *in vivo* in mice.

## Materials and Methods

### Animals and Treatment

Male C57BL/6J mice (8 weeks-old) were purchased from Aisaikesi (Shenyang). The mice were randomly divided into four groups. Sham group, Naringin group, DOX group and Naringin + DOX group. The mice were administered Naringin (50 mg/kg) solution dissolved in 0.5% CMC-Na or 0.5% CMC-Na via oral gavage for 22 days and at seventh day the mice were intraperitoneal injected with Doxorubicin hydrochloride (1 mg/kg) or saline for consecutive 15 days (Figure 1B). At the end of treatment, all mice were intraperitoneally anesthetized to collect the hearts and blood samples for determining heart injury and serum biomarker levels. All procedures were approved by the Animal Care and Use Committee of Dalian Medical University.

### Hematoxylin & Eosin Staining

Hematoxylin & eosin (H&E) staining were carried out according to experiment protocol. Briefly, after the mice hearts were fixed with 4% formaldehyde overnight, they were embedded in paraffin and made into 5 μm sections. The cardiac tissues transversely sectioned from middle segment were stained with H&E for the assessment of myofilament morphology and injury.

### Wheat Germ Agglutinin

For the assessment of the cardiomyocyte cross-sectional area, sections were stained with 50 μg/ml WGA for 60 min, and then it was calculated by measuring 15–20 cells per slid.

### Primary Neonatal Rat Ventricular Myocytes Isolation and H9C2 Treatment

NRVMs were isolated from 1-2-day-old Sprague-Dawley (SD) rats. The heart tissue was cut into pieces and washed with precooled phosphate buffer saline (PBS), and then digested continuously with 0.25% trypsin. The cells were pre-incubated with Dulbecco’s Minimum Essential Medium/F12 (DMEM/F12) containing 10% (vol/vol) fetal bovine serum (FBS) (Life-Lab, AC03L055) and 1% penicillin-streptomycin (PS). Then, the harvested cells were incubated on 100 mm^2^ culture dishes at 37°C with 5% CO_2_. H9C2 cells were obtained from American type culture collection (ATCC). H9C2 cells were cultured in the DMEM containing d-Glucose-(4.5 g/L) with 10% (vol/vol) FBS and 1% PS at 37°C with 5% CO_2._

NRVMs and H9C2 cells were treated with Naringin at a dose of 50 μM in culture medium after cell generation, Doxorubicin hydrochloride was administrated at a dose of 5 μM after Naringin treatment.

### Terminal Deoxynucleotidyl Transferase dUTP NickEnd Labeling Assay

TUNEL Assay kit was used to evaluate cardiac apoptosis according to the manufacturer’s protocol (US EVERBRIGHT, T6014). The apoptotic cells were visualized by fluorescence microscopy and apoptosis was indicated as the ratio of TUNEL-positive nuclei/total stained cell nuclei in five images per heart tissue.

### Catalase, Malondialdehyde, Lactate dehydrogenase, Superoxide Dismutase and Glutathione-Peroxidase Kit Assay

Catalase (CAT), malondialdehyde (MDA), activities of lactate dehydrogenase (LDH), superoxide dismutase (SOD) and Total Glutathione peroxidase (GSH-Px) were measured respectively using assay kits (CAT, MDA, LDH, and SOD kits: Nanjing Jiancheng Company, China; GSH-Px kit: Solarbio, China). The absorbance was detected using EnSpire microplatereader (Tecan, Switzerland).

### Quantitative Real-Time PCR Analysis

Total RNA was isolated from NRVMs or heart tissues using TRIzol (Invitrogen) according to the manufacturer’s protocol. cDNA was used for PCR amplification with gene-specific primers for *cybb* and *nox4*. First-strand cDNA was produced from 1 µg of total RNA from each sample using PrimeScript RT reagent kit according to the manufacturer’s protocol (Yeasen, 11141ES60). The mRNA levels of genes were analyzed using SYBR Green Premix on an Applied Biosystems 7,500 Fast thermocycler. *actn2* was used as the endogenous control.

Table Primer sequences

Gene Forward primer (5′–3′) Reverse primer (5′–3′)

*Cybb* GCT​ACG​CCT​TCA​ACA​CCA​AG AGT​TCG​TCC​CCT​TCT​CCT​GT

*Nox4* CGG​GAT​TTG​CTA​CTG​CCT​CCA​T TGA​CTC​CTC​AAA​TGG​GCT​TCC

*Actn2* ATG​CGG​TTC​CAC​AAG​ATT​GC AGC​CCT​TCT​TTG​GCA​GAT​GTT

### Immunofluorescence Staining

Immunofluorescence staining was performed to detect the expression levels of Bcl-2 (Arigo) and γ-H2AX (abcam) of the cells. After washing with PBS three times, cells were fixed with 4% paraformaldehyde for 30 min, permeated with 0.1% Triton X-100 for 15 min, and then blocked with 3% fetal bovine serum albumin. Cells were incubated with the primary antibodies (anti-Bcl-2, anti-γ-H2AX) overnight at 4°C in a humidified chamber, and then incubated with a green fluorescent secondary antibody (diluted 1:200; Beyotime) for 2 h at room temperature. At last, the slide was staining with DAPI for 10 min and added anti-fluorescence quenching agent. The images were analyzed using a Nikon microscope (Tokyo, Japan).

### MitoSOX Red

MitoSOX Red Assay kit was used to detect the mitochondrial superoxide. H9C2 cells were grown till about 70% confluence and the fresh media were added before experiments. MitoSOX was added to a concentration of 5 μM according to manufacturer’s protocol. Before analysis with confocal microscope, cells were kept 30 min in CO_2_ incubator at 37°C. The digital images were taken by an inverted confocal laser scanning microscope.

### Cell Viability and Cytotoxicity Assay

H9C2 cells were seeded in a 96-well plate and incubated with DOX for 24 h. Cell viability and Cytotoxicity assay was evaluated by Cell Counting Kit-8 (CCK8).

### Western Blot Analysis

H9C2 cells were lysed with radioimmunoprecipitation assay (RIPA) buffer supplemented with PMSF (PMSF: RIPA = 1:100), and the supernatants were collected by centrifugation at 13,300 *g* for 15 min at 4°C. The proteins were separated by 12.5% SDS-PAGE and then transferred to polyvinylidene difluoride (PVDF) membranes by the Bio-Rad Western blotting system. After blocking with 5% non-fat milk for 2 h at room temperature, the membranes were incubated with the primary antibodies (anti-Bcl-2, anti-β-actin) overnight at 4°C and then followed by incubation with secondary antibodies (1:2,500) for 1 h at room temperature. Protein bands were visualized using an ECL kit and Image-Pro Plus 6.0 software was applied to quantify the band intensity values.

### Transfection

In the experiments with si-ECHS1 inhibition, H9C2 cells were divided into five groups: Control, NR (50 μM), DOX (5 μM), NR + DOX (50 μM), NR + DOX (50 μM)+si-ECHS1. si-ECHS1 (5′-3': GGG ACC AUA UCA CCC GGA UTT; 5′-3': AUU CCG GGU GAU AUG GUC CCT T) was transfected with LipFiter 3.0 (Hanbio) for 24 h, followed by subsequent operations.

### Enzyme Linked Immunosorbent Assay

The levels of inflammation factors in heart tissue or H9C2 cells was measured using IL-1β (Elabscience, China), IL-6 (Elabscience, China) and TNF-α (Elabscience, China) ELISA kits according to the manufacturer’s recommendations, The OD is measured spectrophotometrically at a wavelength of 450 nm, the contents of IL-1β, IL-6 and TNF-α were calculated according to the standard curve.

### Data Analysis

Results were expressed as mean ± standard deviation (SD). When the data were normally distributed, the difference between the two groups was analyzed by independent *t*-test, and the comparison between multiple groups was analyzed by one-way ANOVA. Differences were considered to be significant at *p* < 0.05.

## Results

### Naringin Inhibits the Effect of Doxorubicin on Myocardial Atrophy *in vivo*

**FIGURE 2:**
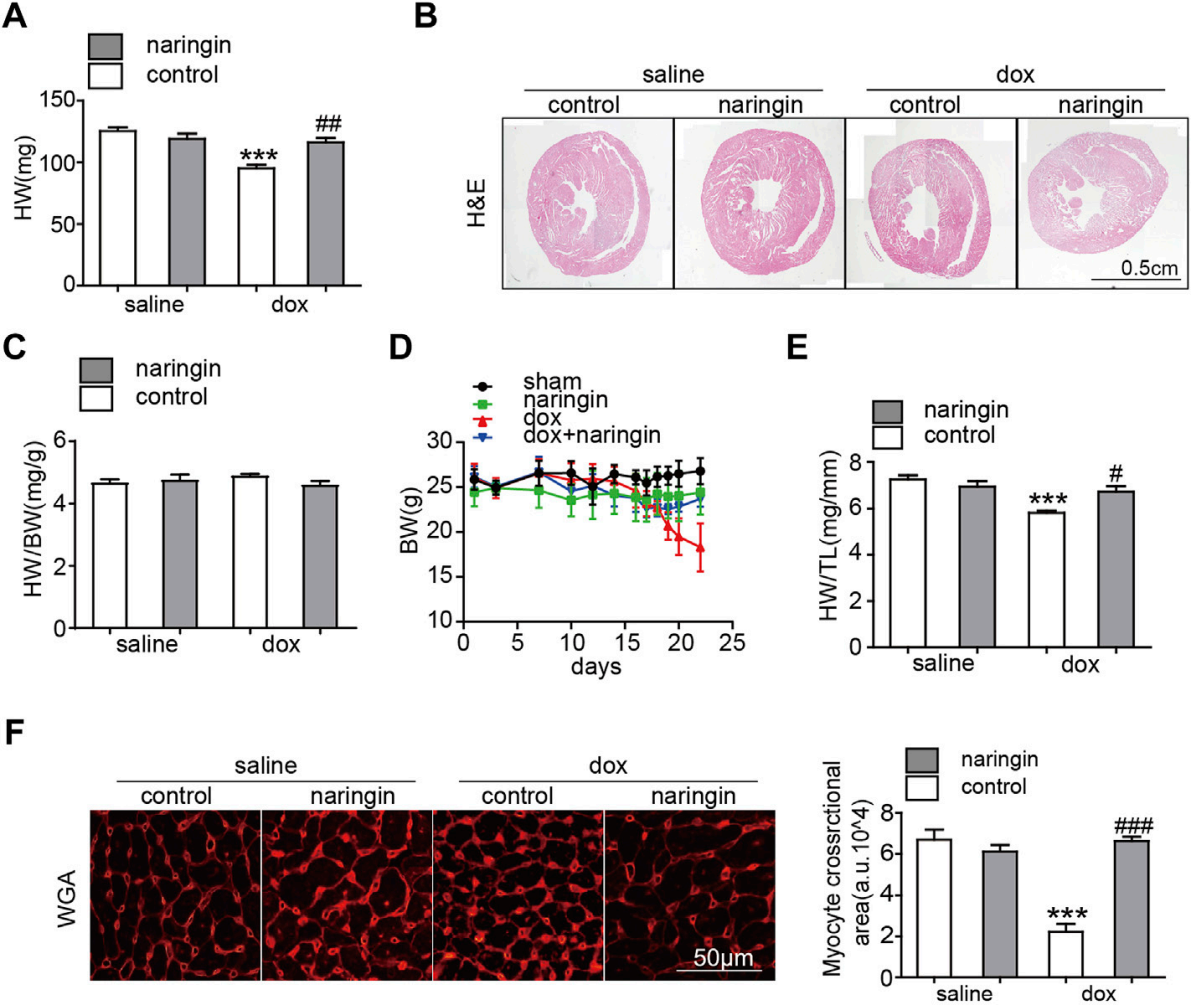
Naringin inhibits the effect of DOX on myocardial atrophy *in vivo*. **(A)** Heart weight of four groups. **(B)** H&E staining of heart sections (Scale bar 1,500 μm). **(C)** Effect of NR on the ratios of heart weight to body weight (HW/BW). **(D)** Body weight of four groups. **(E)** Effect of NR on the ratios of heart weight to tibia length (HW/TL). **(F)** Heart cross-sections were stained with WGA, (left panel). Quantification of the relative myocyte cross-sectional area with quantification analysis (right panel). ***p < 0.001 DOX versus control group; ^#^ *p* < 0.05, ^##^ *p* < 0.01, and ^###^ *p* < 0.001 DOX versus NR + DOX.

To demonstrate the role of Naringin in myocardial injury induced by doxorubicin. Mice were treated Naringin by gavage and then intraperitoneal injection of doxorubicin at a cumulative dose of 15 mg/kg for 2 weeks. The results showed that the heart weight and size of mice treated with doxorubicin significantly decreased, while Naringin increased the heart weight and size of mice treated with doxorubicin (Figures 2A,B). However, the heart weight/body weight ratio of mice did not different among the four groups (Figure 2C), possibly because the body weight of mice also decreased after treatment with doxorubicin (Figure 2D). The tibia length of the mice did not change, so the heart weight/tibia length (HW/TL) results were the same as the heart weight of the mice (Figure 2E). We also did WGA staining to observe the size of myocardial cells in each group. The results showed that myocardial cells in DOX group were significantly smaller, which was consistent with the results in the literature. However, after Naringin treatment, the size of mouse cardiomyocytes increased (Figure 2F). Thus, Naringin could protect the size of mouse cardiomyocytes induced by doxorubicin.

### Naringin Inhibits the Effect of Doxorubicin on Apoptosis, Inflammation and Oxidative Stress *in vivo*

**FIGURE 3:**
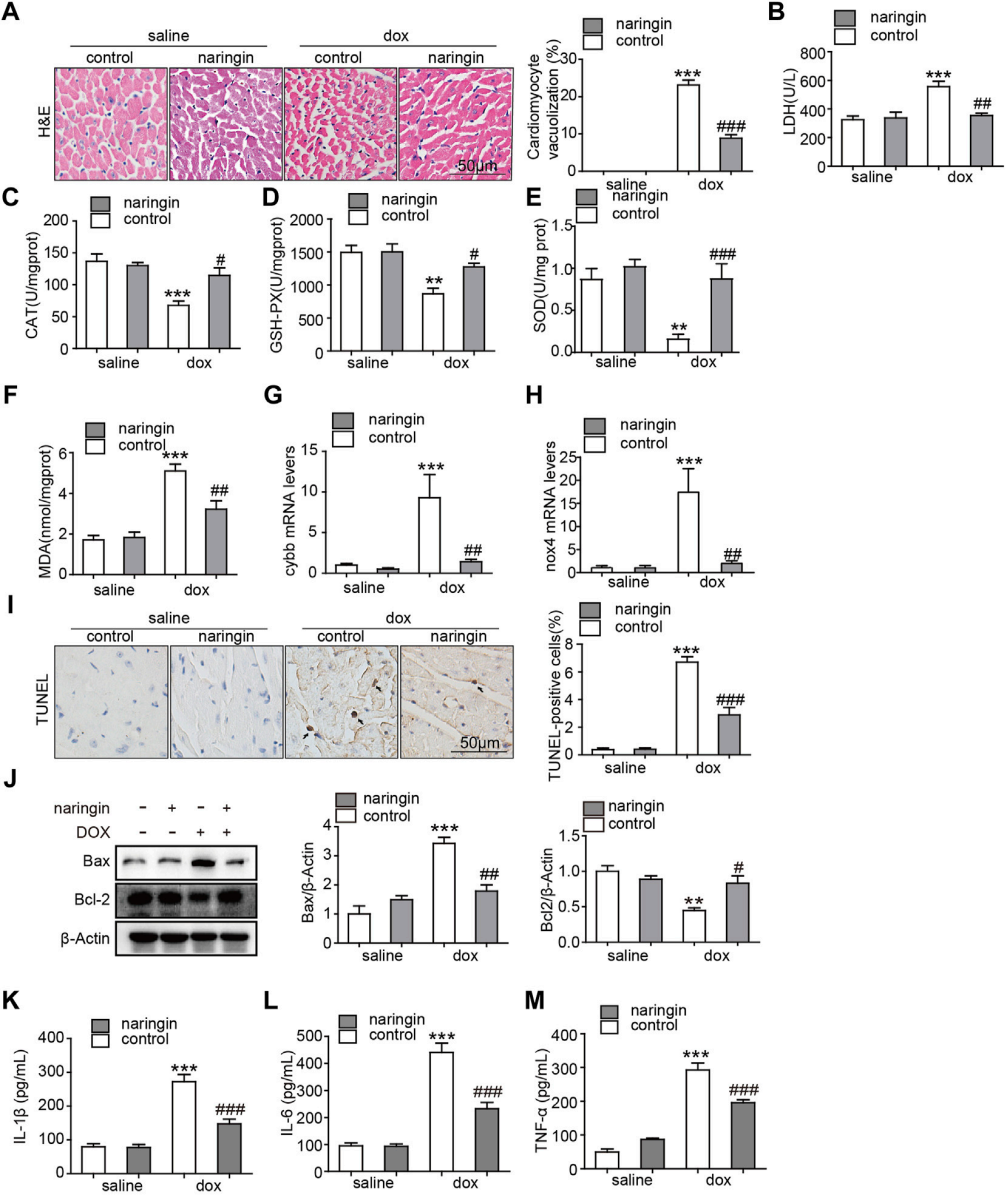
Naringin inhibits the effect of DOX on apoptosis, inflammation and Oxidative stress *in vivo* **(A)** Naringin protects heart from vacuolization induced by DOX. H&E staining of heart sections (left panel), with quantification analysis (right panel). **(B)** The content of LDH (lactic acid dehydrogenase) activity in cardiac. **(C)** The content of CAT activity in cardiac. **(D)** The content of GSH-PX activity in cardiac. **(E)** The content of SOD activity in cardiac. **(F)** The content of MDA activity in cardiac. **(G)** Cardiac mRNA expression level of *cybb* was measured by qPCR analysis. **(H)** Cardiac mRNA expression level of *nox4* was measured by qPCR analysis. **(I)** Mice heart sections were conducted TUNEL staining for determination of apoptotic cells (black arrows indicate TUNEL positive cells, left panel) with quantification of apoptotic cells with quantification analysis (right panel). **(J)** The expression of Bax, Bcl-2 and β-actin detected by Western Blot. **(K**–**M)** The level of IL-1β, IL-6 and TNF-α detected by ELISA. ***p < 0.001 DOX versus control group; ^##^ *p* < 0.01, and ^###^ *p* < 0.001 DOX versus NR + DOX.

There are vacuoles of different sizes in the cytoplasm and nucleus of the denatured cells, and the cells are honeycomb or reticular. Severe denaturation, vesicle mutual fusion into a large vesicle, the nucleus hung in the center, or was squeezed in the side, cytoplasm blank, shape such as balloon. Vacuolation occurs in heart tissue from chemotherapy patients and doxorubicin-induced mice. Here, a large number of vacuolated cardiomyocytes were found in doxorubicin-induced mouse heart tissues by H&E staining, while the number of vacuolated cardiomyocytes was significantly reduced after treatment with Naringin (Figure 3A). Subsequently, we measured the degree of LDH, a marker of myocardial injury. The results showed that doxorubicin promoted the increase of LDH concentration, which was decreased after Naringin treatment (Figure 3B). Doxorubicin could induce apoptosis of cardiomyocytes. Oxidative stress is also a pathway by which cardiomyocytes are damaged by doxorubicin. We detected the concentration level of CAT, GSH-PX, MDA and SOD. They play a crucial role in the balance of oxidation and antioxidant in the organism and is closely related to the occurrence and development of many diseases. In this experiment, the concentration of CAT, GSH-PX and SOD was inhibited by doxorubicin, while the concentration of CAT, GSH-PX and SOD increased after Naringin treatment (Figures 3C–E). The concentration of MDA was increased by doxorubicin, while the concentration of MDA decreased after Naringin treatment (Figure 3F). The mRNA expression levels of oxidative stress related factors NOX2 (cybb) and *nox4* were also detected. The results showed that DOX could significantly promote the expression of cybb and *nox4*, while Naringin inhibited this phenomenon (Figures 3G,H). Next, TUNEL assay was performed to verify whether Naringin has protective effect on doxorubicin-induced apoptosis of cardiomyocytes. The results showed that doxorubicin could indeed induce apoptosis of mouse cardiomyocytes. Naringin, on the other hand, protects cardiomyocytes from apoptosis (Figure 3I). And we examined the marker of apoptosis bcl-2 and Bax by Western Blot. The results showed that Naringin could inhibited the expression of Bax and increase the expression of Bcl-2 induced by DOX (Figure 3J). Local tissue non-specific inflammation caused by DOX chemotherapy. We examined the level of IL-1β, IL-6 and THF-α. The results showed that Naringin could inhibited the level of IL-1β, IL-6 and THF-α induced by DOX (Figures 3K–M). These results suggested that Naringin could reduce cardiac injury by inhibiting apoptosis, inflammation and oxidative stress induced by doxorubicin *in vivo*.

### Naringin Inhibits the Effect of Doxorubicin on Apoptosis, Inflammation and Oxidative Stress *in vitro*

**FIGURE 4:**
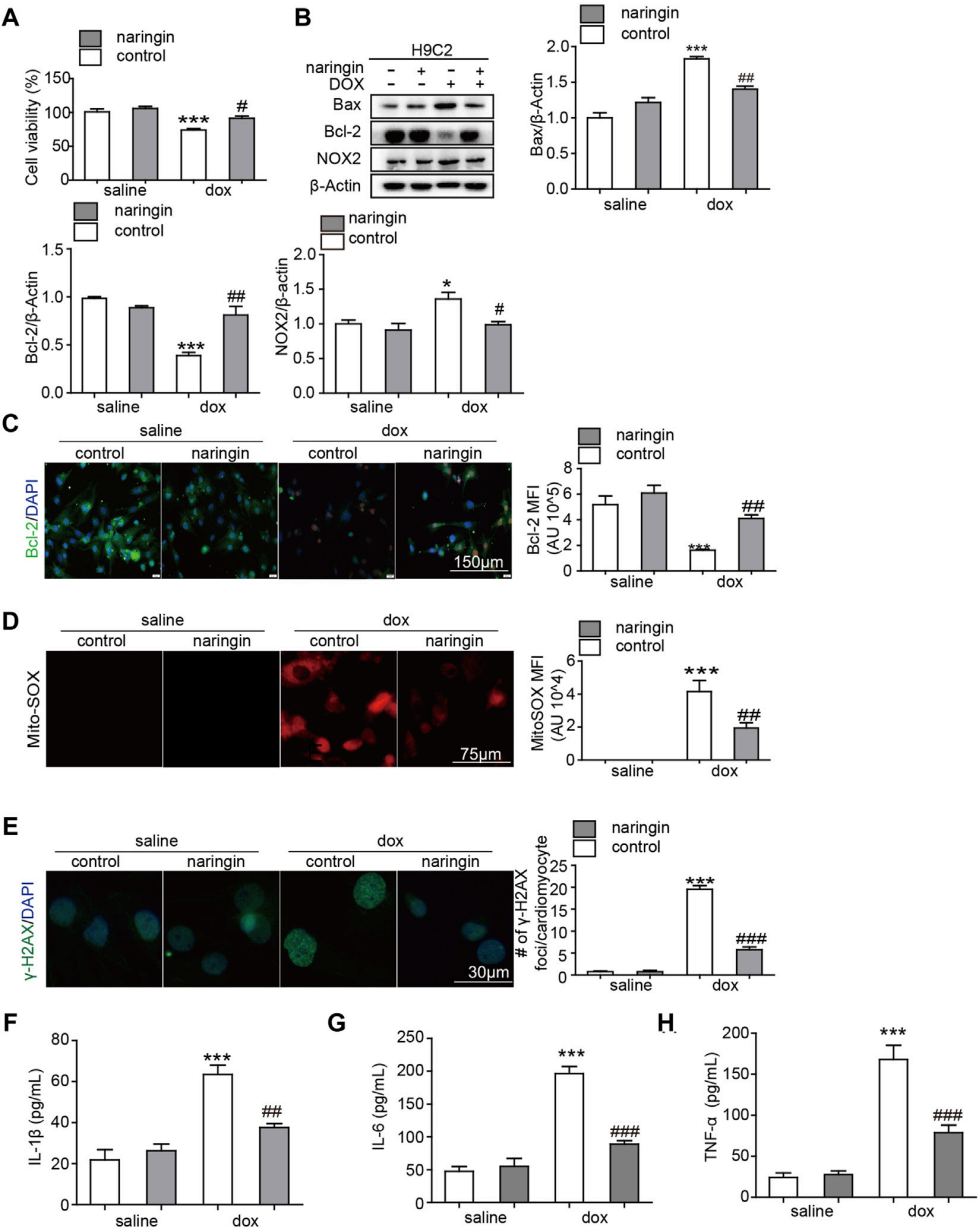
Naringin inhibits the effect of DOX on apoptosis, inflammation and Oxidative stress *in vitro* **(A)** Effect of Naringin on H9C2 cells in the presence of DOX. Cell counting kit 8-based cell viability assays show Cell viability of H9C2 cells in the presence of DOX. **(B)** Effects of Naringin on the protein expression levels of Bcl-2, Bax and NOX2 in H9C2 cells (left panel) with quantification analysis (right panel). **(C)** Naringin treatment increases Dox-Induced Bcl-2 Secretion in NRVMS, which displays representative imaging of bcl-2 immunofluorescence staining. Bcl-2 cells are in green, DAPI in blue. **(D)** Representative Mito-SOX fluorescence images indicate the level of mitochondrial damage (left panel) with quantification of Mito-SOX florescence intensity. **(E)** Representation image of IF staining of the DNA damage markers γ-H2AX (left panel). The average number of γ-H2AX foci in γ-H2AX-positive NRVMS. **(F**–**H)** The level of IL-1β, IL-6 and TNF-α detected by ELISA. ***p < 0.001 DOX versus control group; ^##^ *p* < 0.01, and ^###^ *p* < 0.001 DOX versus NR + DOX.

We have shown that Naringin protects against doxorubicin-induced myocardial damage *in vivo*. Next, we verified this conclusion *in vitro* experiments. First, we used CCK8 assay to test whether Naringin inhibited the cardiotoxicity of doxorubicin. The results showed that doxorubicin could inhibit the proliferation of H9C2 cells and promote their death. Naringin, on the other hand, could protect H9C2 to some extent (Figure 4A). Next, Western blot experiments were used to observe the apoptosis and ROS. The results showed DOX induced the expression of Bax and NOX2, while Naringin inhibited the expression of Bax and NOX2 induced by DOX in H9C2 cells (Figure 4B). And we used IF to detect the expression of bcl-2. The results showed DOX decreased the expression of Bcl-2, while Naringin increased the expression of Bcl-2 induced by DOX in H9C2 cells (Figure 4C). MitoSox™ Red reagent is a novel fluorescent dye that specifically targets mitochondria in living cells. MitoSox™ red reagents are oxidized by superoxide and produce red fluorescence. Next, we used MitoSox™ to observe the effects of DOX and Naringin on oxidative stress. The results showed that the mitochondria of cells induced by doxorubicin showed a large amount of red color, while the red color decreased after treatment with Naringin (Figure 4D). γ-H2AX could detect genomic damage as well as environmental and physical damage caused by cytotoxic chemicals. In this study, doxorubicin could promote the expression of γ-H2AX in the nucleus, while Naringin could inhibit this phenomenon (Figure 4E). We examined the level of IL-1β, IL-6 and THF-α. The results showed that Naringin could inhibited the level of IL-1β, IL-6 and THF-α induced by DOX (Figures 4F,G).

### Inhibition of ECHS1 Interferes the Effect of Naringin on Doxorubicin-Induced on Apoptosis, Inflammation and Oxidative Stress *in vitro*

**FIGURE 5:**
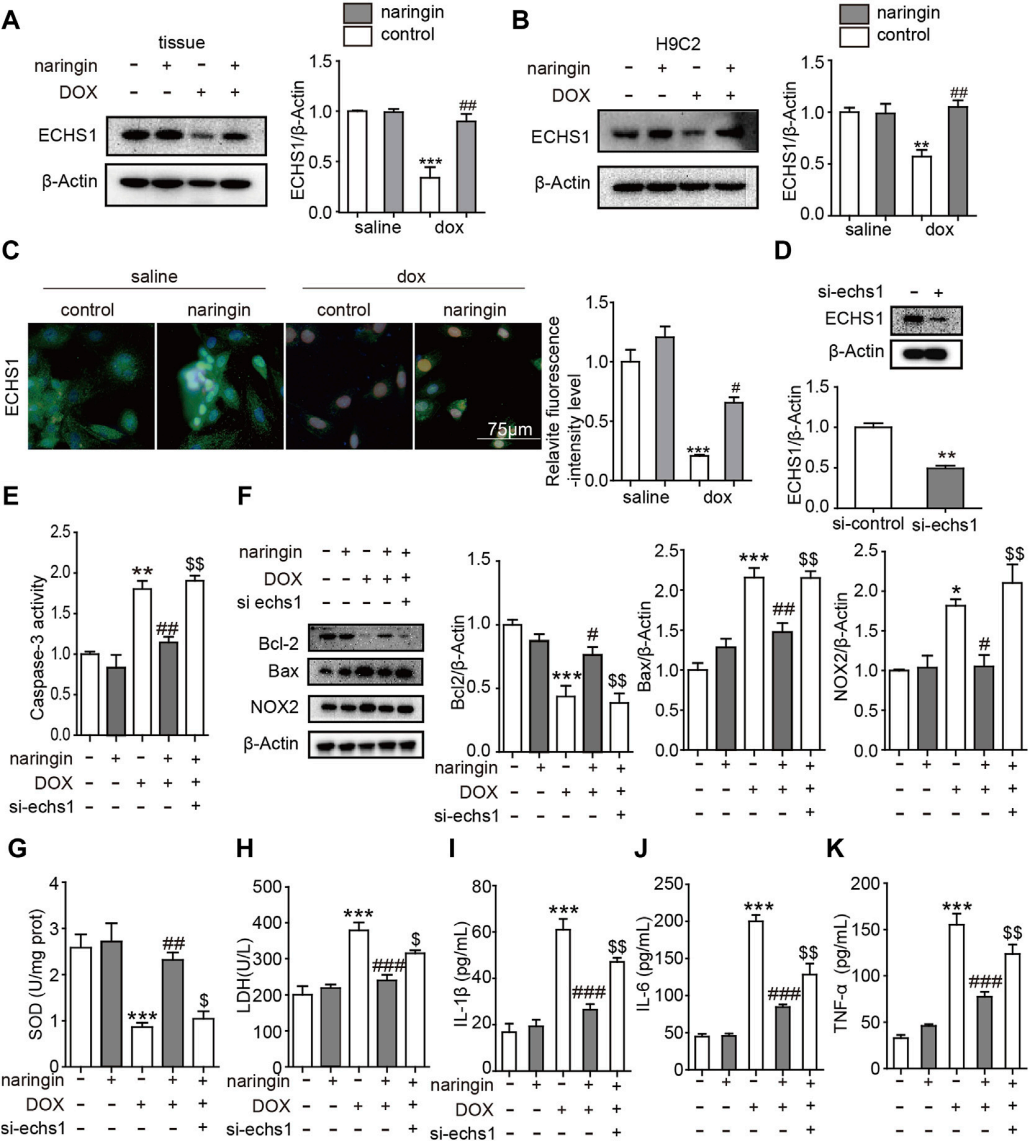
Inhibition of ECHS1 interferes the effect of Naringin on DOX-induced on apoptosis, inflammation and Oxidative stress *in vitro*. **(A)** the protein expression levels of ECHS1 in heart tissue (left panel) with quantification analysis (right panel); **(B)** the protein expression levels of Bcl-2 in H9C2 cells (left panel) with quantification analysis (right panel); **(C)** representative imaging of ECHS1 immunofluorescence staining. ECHS1 cells are in green, DAPI in blue.; **(D)** the protein expression levels of ECHS1 after si-ECHS1 transfection; **(E)** the activity of caspase3 in different group; **(F)** the protein expression levels of Bcl-2, BAX, NOX2 and β-actin in heart tissue (left panel) with quantification analysis (right panel). **(G)** The content of SOD activity in H9C2. **(H)** The content of LDH activity in H9C2. **(I–K)** The level of IL-1β, IL-6 and TNF-α detected by ELISA. **p < 0.01, ***p < 0.001 DOX versus control group or si- ECHS1 versus control group; ^##^ *p* < 0.01, and ^###^ *p* < 0.001 DOX versus NR + DOX; ^$$^ *p* < 0.01 NR + DOX versus NR + DOX + si- ECHS1.

We have proved Naringin inhibits the effect of DOX on apoptosis, inflammation and Oxidative stress *in vitro*. Next we looked at what proteins were involved. Oxygen free radicals produced by DOX cause cell damage and could peroxidize unsaturated fatty acids. ECHS1 mutations lead to decreased fatty acid mitochondrial β-oxidation activity, thereby reducing the formation of important energy substrates (such as acetyl CoA), reducing the production of ATP, leading to energy deficiency in the catabolic state (such as fever, after viral infection), and increasing organs. The susceptibility to dysfunction is especially obvious in tissues and organs that rely on fatty acids and ketone bodies as energy sources (such as heart tissue) (Haack et al., 2015). So we guess whether ECHS1 plays a role in the above results. First of all, we verified that DOX could inhibit the expression of ECHS1 in tissues and cells, and after treatment with Naringin, the expression of ECHS1 increased significantly (Figures 5A–C). Next, we constructed the siRNA of ECHS1, and verified by WB that ECHS1 did interfere with the sequence effectively (Figure 5D). Finally, we transfected si-ECHS1 in the presence of both DOX and Naringin, and found that after transfection of si-ECHS1, the activity of caspase3 increased (Figure 5E), the expression of apoptosis-related protein bcl-2 decreased, the expression of BAX increased, and the expression of oxidative stress-related protein NOX2 increased (Figure 5F). The concentration of SOD was inhibited by DOX, while the concentration of SOD increased after Naringin treatment. However, the level of SOD was decreased again after ECHS1 knockdown (Figure 5G). Then we measured the degree of LDH. The results showed that DOX promoted the increase of LDH concentration, which was decreased after Naringin treatment. However, the level of LDH was increased again after ECHS1 knockdown (Figure 5H). We examined the level of IL-1β, IL-6 and THF-α. The results showed that Naringin could inhibited the level of IL-1β, IL-6 and THF-α induced by DOX. However, the level of IL-1β, IL-6 and THF-α was increased again after echs1 knockdown (Figures 5I–K).

## Discussion

Anthracyclines are currently the most widely used anticancer drugs (Martins-Teixeira and Carvalho, 2020). As its classic representative, DOX has significant dose cumulative cardiotoxicity, which could eventually lead to heart failure with a poor prognosis and a 2-years mortality of up to 50% without treatment (Cardinale et al., 2020). Currently, there is no specific protocol to prevent DOX-induced cardiotoxicity. To standardize the treatment of heart failure caused by DOX, reduce the symptoms, improve the quality of life, and find a drug will have important clinical significance (Rochette et al., 2015).

**FIGURE 6:**
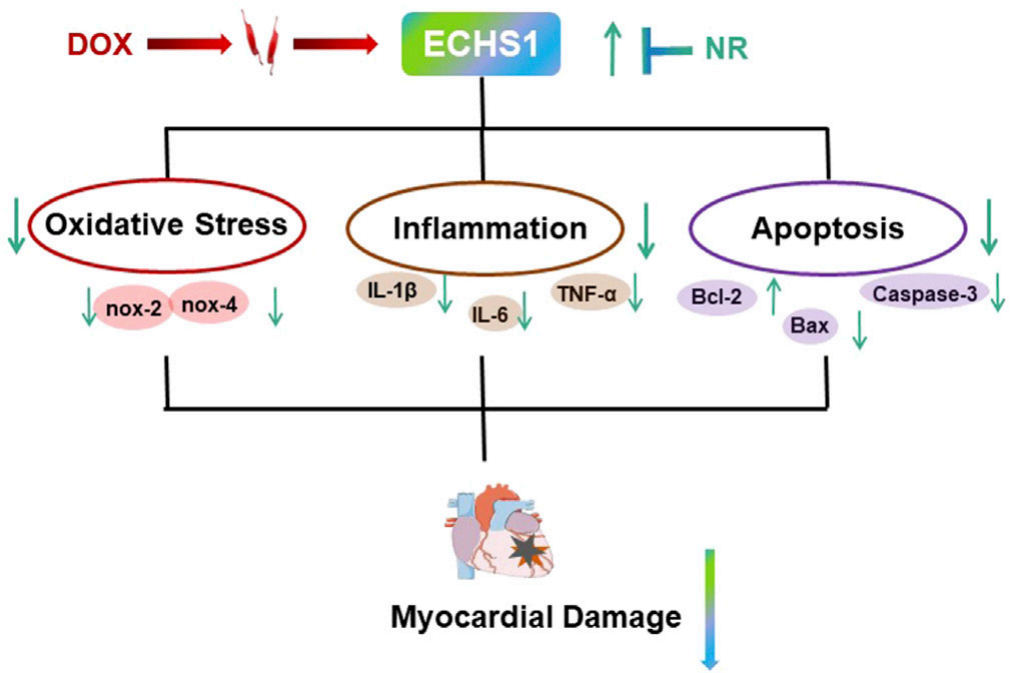
Schematic diagram of the mechanism.

In this study, we investigated the role of Naringin in myocardial injury induced by DOX in mice (Figure 6). Naringin has been extensively studied in cancer, rheumatic disease and a variety of cardiovascular diseases (Bai et al., 2019; Heidary et al., 2020; Yang et al., 2020), and its main mechanism is to protect cells from oxidative stress (Zhou et al., 2019) and apoptosis (Zhao et al., 2020). The pathogenic mechanism of doxorubicin is also the induction of oxidative stress and apoptosis (Hu et al., 2020; Zhang et al., 2020). The results show that Naringin did protect against DOX-induced myocardial injury, oxidative stress, and apoptosis. Naringin could increase HW/TL ratio and inhibit LDH level. Mitochondria are the centers of cell energy metabolism. Mitochondria are especially abundant in the sarcoplasm of myocardium (Cai et al., 2015), which adapts to the characteristics of continuous rhythmic contraction of myocardium. So the heart is also more vulnerable to oxidative stress. The results in this paper were the same as those reported, DOX increased oxidative stress level, decreased SOD level and increased mRNA level of oxidative stress related factors in mouse myocardium. However, Naringin could promote the increase of SOD level and reduce the mRNA level of oxidative stress related factors. So Naringin reduced the level of DOX-induced oxidative stress, which was one of the reasons for protecting the mouse heart from damage.

Apoptosis of cardiomyocytes is also one of the causes of heart damage (Imam et al., 2018; Zhou et al., 2021). In this study, we found that DOX could promote apoptosis of cardiomyocytes, inhibit cell activity, and induce vacuolation. This result was consistent with previous studies. After Naringin treatment, the number of apoptosis and vacuolation of myocardial cells decreased. The expression levels of apoptosis-related proteins were decreased.

IL-6 is a pleiotropic cytokine, which is related to pro-inflammatory and anti-inflammatory effects. TNF-α and IL-1β are key pro-inflammatory cytokines involved in the induction of inflammatory response. Inflammation is an important mechanism that triggers DOX-induced myocardial damage. In this article, we tested the expression levels of three cytokines to prove that NR could regulate DOX-induced inflammation through ECHS1.

ECHS1 encodes for short-chain enoyl-CoA hydratase, a key component in b-oxidation. This enzyme is also involved in the isoleucine and valine catabolic pathways. Its lack of clinical manifestations include severe psychomotor developmental delay, epilepsy, neurodegeneration, dystonia, respiratory insufficiency, metabolic acidosis, and feeding difficulties. In this study, we found that DOX could inhibit the expression of ECHS1. Naringin could promote the expression of ECHS1 induced by DOX. And after interfering with ECHS1, the levels of cell apoptosis, inflammation and oxidative stress showed a tendency to increase again.

Evidence suggests that Naringin couldimprove gentamicin-induced renal toxicity and related mitochondrial dysfunction, apoptosis and inflammation in rats, and inhibit fructose production of mitochondrial ROS and mitochondrial dysfunction in cardiomyocytes (Sahu et al., 2014). Naringin reduces cardiac hypertrophy by regulating ampK-MTOR signaling pathway (Park et al., 2018). Studies have shown that Naringin inhibits cardiomyocyte apoptosis induced by high glucose by reducing mitochondrial dysfunction and regulating the activation of P38 signaling pathway (Huang et al., 2013). These studies suggest that Naringin has a protective effect on diseases by inhibiting mitochondrial dysfunction.

ECHS1 is a multifunctional mitochondrial matrix enzyme involved in the oxidation of essential amino acids such as fatty acids and valine. Lack of ECHS1 leads to mitochondrial encephalopathy (Haack et al., 2015), and ECHS1 plays an important role in maintaining mitochondrial function.

Therefore, given the critical roles of Naringin and ECHS1 in maintaining mitochondrial function, we hypothesized that ECHS1 plays a role in Naringin against DOX-induced myocardial injury.

Because Naringin is a dihydroflavone compound, which exists in natural products, there was no difference between the Naringin group and the control group after the mice were given intragastric gavage. So Naringin has the potential of clinical application.

## Data Availability

The raw data supporting the conclusions of this article will be made available by the authors, without undue reservation.
